# Effect of Fermentation with Different Lactic Acid Bacteria Starter Cultures on Biogenic Amine Content and Ripening Patterns in Dry Fermented Sausages

**DOI:** 10.3390/nu10101497

**Published:** 2018-10-13

**Authors:** Federica Pasini, Francesca Soglia, Massimiliano Petracci, Maria Fiorenza Caboni, Sara Marziali, Chiara Montanari, Fausto Gardini, Luigi Grazia, Giulia Tabanelli

**Affiliations:** 1 Centro Interdipartimentale di Ricerca Industriale Agroalimentare, Alma Mater Studiorum, Università di Bologna, Sede di Cesena, Piazza Goidanich 60, 47521 Cesena, Italy; federica.pasini5@unibo.it (F.P.); m.petracci@unibo.it (M.P.); maria.caboni@unibo.it (M.F.C.); chiara.montanari8@unibo.it (C.M.); fausto.gardini@unibo.it (F.G.); giulia.tabanelli2@unibo.it (G.T.); 2 Dipartimento di Scienze e Tecnologie Agro-alimentari, Alma Mater Studiorum, Università di Bologna, Sede di Cesena, Piazza Goidanich 60, 47521 Cesena, Italy; 3 Dipartimento di Agricoltura, Ambiente e Alimenti, Università degli Studi del Molise, via De Sanctis snc, 86100 Campobasso, Italy; s.marziali@studenti.unimol.it; 4 Dipartimento di Scienze e Tecnologie Agro-alimentari, Alma Mater Studiorum, Università di Bologna, Sede di Bologna, Viale Fanin 44, 40127 Bologna, Italy; luigi.grazia@unibo.it

**Keywords:** fermented meat, starter cultures, biogenic amines, sausage size, proteolysis, lipolysis

## Abstract

In the present study, two different diameter (small and large) Milano-type dry fermented sausages were industrially produced to evaluate the effect of two different LAB starter cultures (*Lactobacillus sakei* and *Pediococcus pentosaceus*) on biogenic amines (BAs) content, proteolysis, and lipolysis taking place during both fermentation and ripening. With regard to BAs, putrescine and tyramine were mostly found in fermented sausages having large diameter and those inoculated with *P. pentosaceus*/*S. xylosus* exhibited significantly higher accumulation of these compounds. Overall, the small size sausages showed a more pronounced proteolysis taking place during processing. In addition, aside from the distinctive electrophoretic bands detected with both starter cultures, a more pronounced proteolysis and a faster protein hydrolysis was observed in salami inoculated with *P. pentosaceus*/*S. xylosus*. As for lipolysis, a significantly higher amount of diacylglycerols was observed at the end of ripening in the sausages inoculated with *L. sakei*/*S. xylosus*, which concurrently exhibited an increased D32, D34, and D36 series. The results of the present study confirms profound differences in BAs concentration, proteolysis, and lipolysis. These findings are strictly dependent on the starter cultures, which demonstrates that the choice of an appropriate starter optimized for peculiar products and processes should be the key factor to improve safety and quality features of traditional fermented sausages.

## 1. Introduction

Fermented sausages are a heterogeneous group of products differentiated in relation to raw material (type of lean meat and fat), the mincing degree of meat batter, ingredients (salt concentration, nitrate/nitrite, spices and herbs, other additives), size (diameter and weight), type of casing, starters, and ripening conditions (temperature, relative humidity, use of moulds, and/or smoke) [1,2]. After casing, several modifications occur during fermentation and ripening, which bring to the acquisition of the organoleptic and textural properties desired for each type of sausage. These modifications are influenced by physico-chemical factors due to the action of salt on the formation of a gel structure and autoxidation reactions and biochemical activity due to the endogenous enzymes of the meat and microbial activities [3]. The solubilization and gelification of myofibrillar proteins exert an essential role in the formation and development of fermented sausages’ structure. In detail, this process is widely influenced by the salt concentration within the meat batter and the decrease of pH following lactic fermentation [4,5]. In this context, in fermented sausages, proteolysis is one of the main phenomena taking place during ripening [6]. Meat proteins are the target of enzymes such as peptidases and proteases such as calpains and cathepsins (in particular cathepsin D), which catalyse the hydrolysis of the myofibrillar proteins first to polypeptides and, subsequently, to smaller peptides leading in the final step to the formation of free amino acids [7,8]. Moreover, sausage microbiota actively contributes to protein hydrolysis and peptide formation especially during ripening. This proteolytic activity has been widely studied in staphylococci and moulds even though a role of lactic acid bacteria (LAB) has been demonstrated [9,10,11]. Afterward, free amino acids and small oligopeptides can be further metabolized by microorganisms resulting in energy production (i.e., arginine metabolism), development of flavor compounds (for branched chain or aromatic amino acids) and accumulation of biogenic amines (BAs) [12,13,14]. These latter compounds can be present in food, which causes several adverse reactions in the consumers. In fact, ingestion of food containing high amounts of biogenic amines is implicated in the headache, heart palpitations, vomiting, and diarrhea since histamine and tyramine are the most dangerous and are responsible for symptomatology known as “scombroid fish poisoning” and “cheese reaction,” respectively [15]. The presence of BAs in food is dependent both on food contamination by decarboxylating microorganisms on the precursor availability (proteins and/or free amino acids) and on different intrinsic, environmental, and technological factors. In fermented sausages, the presence of high precursor concentrations and of decarboxylase positive non-starter microflora during the ripening period can favor a high accumulation of these compounds (tyramine and putrescine) [16]. In these products, since BA reduction is often limited by the fermentation and ripening conditions, the main tool to counteract their accumulation is the choice of appropriate starter cultures able to rapidly and persistently colonize the meat batter and have the capacity to inhibit or reduce the growth of aminobiogenetic wild microorganisms (i.e., with bacteriocin producing strains) [15]. 

Alongside proteolysis, lipolysis plays a fundamental role during ripening. Endogenous lipase and esterase produce free fatty acids, precursors for oxidative transformations bringing the formation of compounds (alkanes, alkenes, aldehydes, alcohols, ketones) with a strong impact on the aroma profile of sausages. In addition, several authors underlined a possible role of microbial lipases (and esterases) produced by molds and staphylococci [17]. All these transformations contribute to the formation of the “typical” characteristics, which are associated to each sausage typology and concern physico-chemical, textural, and sensorial features as well as product safety [18]. It has been reported that sausage diameter plays a relevant role in the formation of the desired characteristics. In fact, it affects many important events during ripening such as the removal of water and the presence of oxygen inside the sausage, which, in turn, influence the biochemical activities of this crucial period [19,20,21]. Recently, some authors applied a linear discriminant analysis to highlight the role of diameter on the aroma profile and chemico-physical characteristics of different Italian industrial fermented sausages typologies [20,21].

Among the main drivers of the complex phenomena, which take place during ripening, the use of starter cultures together with rigorous temperature and relative humidity conditions are the main tools adopted by the fermented sausage industry to improve the quality and safety of its products [22]. In Italy, the use of selected starter cultures [consisting in lactic acid bacteria (LAB), staphylococci, and molds] is nowadays widespread in industrial products [23,24]. However, in many cases, the introduction of this technological practice has not been accompanied by deeper studies of starter fermentation impact on the sausage characteristics and health features in relation to the type of process and product. 

In this work, two different diameter Milano-type dry fermented sausages were industrially produced to evaluate the effects of fermentation performed by two different LAB starter cultures (*Lactobacillus sakei* and *Pediococcus pentosaceus*) on the presence of BAs during the production and the ripening. Moreover, lipolytic and proteolytic activities during fermentation, maturation, and in the final products were assessed to understand the role of LAB in ripening patterns. For the same production, microbiota evolution (both by culture-dependent and culture-independent methods), physico-chemical parameters and aroma profile were evaluated. The results have been recently published [25].

## 2. Materials and Methods 

### 2.1. Sausage Manufacturing

**Figure 1:**
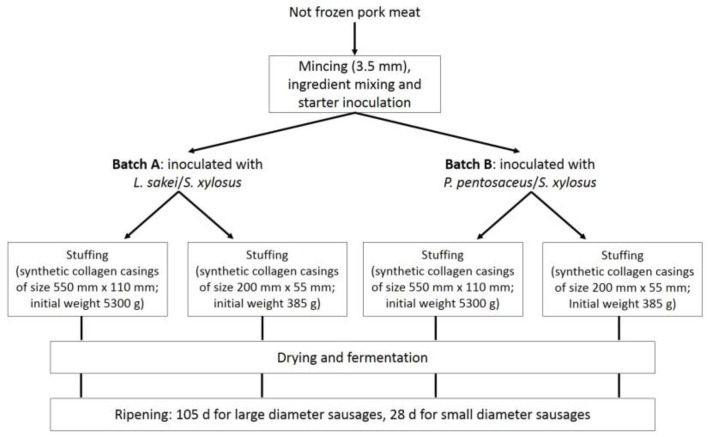
Process scheme of the manufacturing of the Milano sausages produced with the two starter cultures.

The Milano-type dry fermented sausages used in this study were manufactured in C.l.a.i. Soc. Coop. (Imola, Italy) following the same process reported by Reference [25]. For each condition tested, sausages were produced from a batch of approximately 600 kg based on the industrial usual procedures and the ripening period required by the two different sausage sizes studied. The same meat mixture (containing pork shoulder (72% *w*/*w*), streaky bacon (28% *w*/*w*), NaCl (2.6% *w*/*w*), dextrose (0.30% *w*/*w*), KNO_3_ (0.015% *w*/*w*), NaNO_2_ (0.010% *w*/*w*), garlic, and white and black pepper powder (0.12% *w*/*w*) were divided into two aliquots and inoculated with the two different starter cultures provided by Chr. Hansen (Parma, Italy). The batch named A was inoculated with *Lactobacillus sakei* and *Staphylococcus xylosus* while the batch named B with *Pediococcus pentosaceus* and the same staphylococcal starter have an initial concentration of LAB and staphylococci of approximately 6 log cfu/g. Each aliquot was stuffed in two synthetic casings with different diameters and lengths (Figure 1). 

A spore suspension of the *Penicillium nalgiovense* strain was sprayed on the casings. The samples were collected during the manufacturing process and during ripening: immediately before casing, after 3 days, and after 5 days of fermentation, at approximately 30%, 60%, and 100% of ripening time (corresponding to 38, 69, and 105 days for large diameter sausages and 11, 17, and 28 days for small diameter sausages). 

All the analyses were run in triplicate and the results were expressed as a mean value of three independent samples (three different sausages).

### 2.2. Weight Losses and pH

Each sample has been weighed during the production and ripening period to calculate the mean weight loss (%) with respect to the initial one, which was about 5300 g for the big diameter sausages and 385 g for the small ones.

### 2.3. Biogenic Amine

For the detection of biogenic amines content, three sausage samples were extracted with trichloroacetic acid, according to Coloretti et al. [26]. The extracts were subjected to a dansyl chloride derivatization (Sigma Aldrich, Gallarate, Italy), according to Martuscelli et al. [27]. The biogenic amines content was analyzed by using a PU-2089 Intelligent HPLC quaternary pump and an Intelligent UV-VIS multi wave length detector UV 2070 Plus (Jasco Corporation, Tokio, Japan) following the method reported by Gardini et al. [28]. The amounts of amines were expressed as mg/L by reference to a calibration curve obtained with aqueous biogenic amine standards derivatized as described for the samples. 

### 2.4. Proteolysis of Myofibrillar Proteins

Proteolysis was assessed by evaluating the electrophoretic profile of the myofibrillar proteins extracted, according to the procedure described by Hughes et al. [29], from meat batters immediately before casing, after 5 days of fermentation, and at the end of the ripening process. One gram (in duplicate) of frozen meat batter was homogenized (30 sec at 13,000 rpm in ice) in 20 mL of cold Rigor Buffer (75 mM KCl, 10 mM KH_2_PO_4_, 2 mM MgCl_2_, 2mM EGTA, pH 7.0). The homogenate was centrifuged for 10 min at 10,000× *g* at 4 °C and the supernatant discarded (sarcoplasmic protein). The procedure was repeated and the resultant pellet, identified as the myofibrillar protein fraction, was re-suspended by homogenization in 20 mL of cold Rigor Buffer. After being quantified [30], the protein concentration of each extract was adjusted to 1.0 mg/mL and each sample was mixed 1:1 (*v*/*v*) with Sample Buffer (50 mM Tris-HCl, 8 M Urea, 2 M Thiourea, 75 mM DTT, 3% (*v*/*v*) SDS; pH 6.8). SDS-PAGE analysis was run, in duplicates, on 5 μg of proteins, according to the procedure described by Laemmli et al. [31] by using 7.5% polyacrylamide hand-cast gels. A molecular weight marker (Precision Plus Standard Proteins, All Blue Prestained, Bio-Rad, Hercules, CA, USA) was loaded into each gel to assess the molecular weight of the protein bands. The gels were run on a Bio-Rad Mini Protean II electrophoresis apparatus at 110 V constant voltage for about 1 h. The gels were stained with Coomassie Brilliant Blue R-250 (1 g/L) containing 40% (*v*/*v*) methanol and 10% (*v*/*v*) acetic acid in distilled water and de-stained in distilled water. The gel images were acquired by using a GS-800™ Calibrated Densitometer (Bio-Rad) to quantify the relative abundance of each protein band. 

### 2.5. Lipid Characterization

Fatty acid (FA) composition, diacylglycerols (DAGs), peroxide value (PV), and TBARS were carried out to control lipidic changes during the fermentation/ripening process and in the final products.

The lipids were extracted, according to a modified version of the method described by Folch et al. [32] and approximately 20 mg of fat were used for transesterification to the corresponding methyl esters (FAMEs) by following the method of Christie et al. [33]. FAMEs were measured on a GC-2010 Plus gas chromatograph (Shimadzu Corporation, Kyoto, Japan) equipped with a flame ionization detector (FID), according to the method of Ben Lajnef [34]. The FAME peaks were separated with a BPX70 fused silica capillary column (10 m, 0.1 mm i.d., 0.2 m f.t.), coated with 70% cyanopropyl polysilphenylenesiloxane film from the SGE Analytical Science (Melbourne, Australia), and identified by comparison of the retention times with fatty acid methyl ester GLC-463 from Nu-Check (Elysian, MN, USA) and FAME 189-19 standard mixtures from Sigma-Aldrich Chemicals (St. Louis, MO, USA). FAs were quantified by comparing the peak area of each compound with that of IS (Methyl tridecanoate (C13:0; 1 mg/mL) and expressed as mg of FAME/100 mg of fat.

DAG were purified by silica SPE, which was suggested by Bortolomeazzi et al. [35]. Approximately 70 µL of internal standard (1 mg/mL of 5α-colestan-3β-ol) was added to the fat sample before the SPE purification and the final DAG fractions were silylated to the corresponding trimethylsilyl (TMS) ethers, according to Sweeley et al. [36]. One microliter of the silylated solution was injected into GC by using a fused-silica capillary column (30 m × 0.25 mm i.d. × 0.1 µm film thickness) coated with 65% diphenyl and 35% dimethylpolysiloxane (Rtx 65TG; Restek). The injector and detector temperatures were set at 350 °C. The oven temperature was set at 240 °C for 0.50 min, raised to 350 °C at a rate of 10 °C/min, and kept at 350 °C for 15 min. The carrier gas (He) flow rate was 3.84 mL/min and the split ratio was 1:100.

The content of hydroperoxides was estimated by determining the peroxide value of lipids expressed as meq of O_2_ per kg of fat, according to Shantha and Decker [37].

Thiobarbituric acid-reactive substances (TBARS) used as an index of lipid oxidation were measured in duplicates by following the procedure described by Bao and Ertbjerg [38] and the results expressed as mg MDA/kg of meat.

### 2.6. Statistical Analysis

Three independent different sausages were investigated for each sampling time with each analyzed in triplicate. Potential interactions between factors (sausage size, starter cultures, and process time) were explored by the general linear model (GLM) procedure using three-way ANOVA of Statistica (StatSoft Italy srl, Vigonza, Italy). Fisher’s Least Significant Difference (LSD) test was used to identify significant differences (*p* < 0.05).

A Principal Component Analysis (PCA) was carried out by using variables, which resulted as significantly different on the basis of the statistical analysis reported above. All statistical analyses were carried out by using Statistica 8.1 (StatSoft Italy srl, Vigonza, Italy).

## 3. Results

### 3.1. Weight Losses

**Figure 2:**
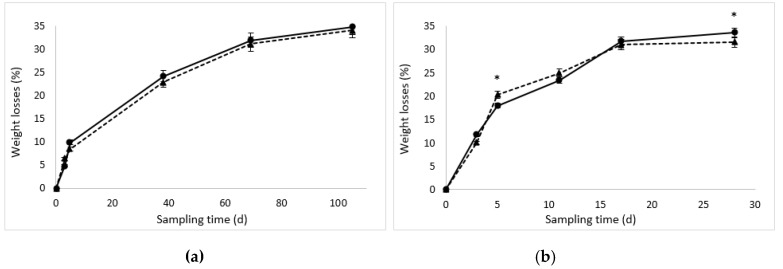
Weight losses (%) with respect to the initial weight during fermentation and ripening of the sausages inoculated with the two different starters: (**a**) large diameter and (**b**) small diameter. The dotted line with the triangles represents the inoculated samples with *L. sakei*/*S. xylosus* while the continuous line with circles represents the inoculated samples with *P. pentosaceus*/*S. xylosus*. An asterisk indicates the presence of significant differences between starters for each sausage size and each sampling time.

The water losses during ripening determined a progressive diminution of sausage weight (Figure 2). Since sausage weight loss is dependent on product size and ripening, which determine different water migration in the mass, one-way ANOVA was used for each product type to determine the statistically significant difference in relation to the starter culture employed. At the end of ripening, the large diameter sausages lost about 34% of weight without significant differences depending on the starter cultures. In the small sausages, the final weight loss differed significantly (*p* < 0.05) in relation to the starting cultures being 31.6% in the samples inoculated with *L. sakei*/*S. xylosus* and 33.4% in those inoculated with *P. pentosaceus*/*S. xylosus*. These weight losses corresponded to a final a_w_ value of 0.912 in the large diameter sausages and of 0.924 and 0.916 in the small samples inoculated with *L. sakei*/*S. xylosus* and *P. pentosaceus*/*S. xylosus*, respectively [25]. 

### 3.2. Biogenic Amine Content

BA concentrations in meat batter immediately before casing, during ripening, and in the final products are reported in Table 1. 

Their content highly differed in relation to the product diameter due to the overall BA content in small size sausages being less than 25 mg/kg at the end of ripening. Histamine and cadaverine were detected sporadically in negligible amounts (<3 and <10 mg/kg, respectively). Putrescine was occasionally detected in the small diameter sausages while relevant differences were observed in relation to the starter culture used in the large diameter sausages. In fact, in the samples inoculated with *L. sakei*/*S. xylosus*, putrescine concentration was lower than 10 mg/kg even at the end of ripening while a significantly higher accumulation of this BA was found in the sausages inoculated with *P. pentosaceus*/*S. xylosus*. In the final product of these samples, the concentration of putrescine was about 128 mg/kg. Tyramine was detected in irrelevant amounts in the small diameter sausages. Conversely, in the large diameter products, its accumulation was higher than 100 mg/kg after 38 days of ripening and reached significantly higher concentrations in the samples inoculated with *P. pentosaceus*/*S. xylosus* if compared with those in which *L. sakei*/*S. xylosus* was added (204 vs. 177 mg/kg).

### 3.3. Proteolysis of Myofibrillar Proteins

**Figure 3:**
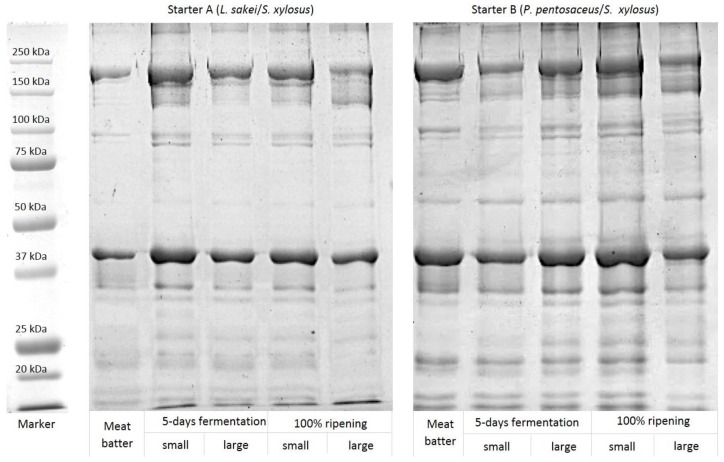
SDS–Polyacrylamide gel electrophoretic patterns of myofibrillar proteins for sausages inoculated with different starters (*L. sakei*/*S. xylosus* and *P. pentosaceus*/*S. xylosus*) and have different diameters (large and small) during fermentation and ripening.

In the present study, the electrophoretic profiles of the myofibrillar proteins extracted from meat batters before casing and from sausages after five days of fermentation and, at the end of the ripening process, the electrophoretic profiles were inoculated with a different starter (*L. sakei*/*S. xylosus* and *P. pentosaceus*/*S. xylosus*) and had different diameters (large and small), are shown in Figure 3. 

Overall, the greatest changes were observed in contractile and cytoskeletal proteins as well as in those polypeptide chains composing the tropomyosin complex (Table 2). The results were expressed as relative abundance (%) in order to minimize the differences resulting from loading.

A significant reduction in the relative intensities of the bands ascribed to Myosin Heavy Chain (MHC), desmin, actin, tropomyosin, troponin C, and MLC2 was observed during both fermentation and ripening. Concurrently, protein hydrolysis was associated with the simultaneous appearance of several degradation products with a molecular weight ranging from 50 to 150 kDa. As an example, MHC degradation results in the accumulation of a 110 kDa polypeptide. In spite of a progressive degradation after fermentation, troponin T, troponin C, and tropomyosin resulted in more intense staining after ripening. In addition, aside from the starter cultures, differences in the proteolytic patterns were observed by comparing salami with different diameters. If compared to large ones, those with a small size exhibited a more pronounced proteolysis taking place during fermentation and subsequent maturation of the product, which is shown by a high number of polypoptide fragments in an SDS-PAGE electoforetic gel. In addition, in small size sausages, Troponin C was significantly affected by starter cultures. In fact, in sausages inoculated with *L. sakei*/*S. xylosus*, a more intense degradation of this protein was observed at the end of ripening. Two polypeptides with a molecular weight of 95.8 and 36.1 kDa were identified in salami inoculated with *L. sakei*/*S. xylosus*. The last one was mainly found in the small diameter ones. On the other hand, distinctive electrophoretic bands of 191, 174, and 150 kDa were observed in salami inoculated with *P. pentosaceus*/*S. xylosus* that exhibited a more pronounced proteolytic activity, which was only marginally affected by the diameter, and a faster degradation of the polypeptides was compared to those in which *L. sakei*/*S. xylosus* were added.

### 3.4. Lipid Fraction

Fatty acid (FA) composition, diacylglycerols (DAGs) peroxide value (PV), and TBARS of the sausage samples obtained with a different diameter (large and small) and a different starter (*L. sakei*/*S. xylosus* and *P. pentosaceus*/*S. xylosus*) were analyzed during processing (meat batters, after five days of fermentation, and at the end of maturation) and the results are reported in Table 3.

The total fat content in sausage samples increased gradually throughout ripening, according to water loss (Figure 1). The initial fat content was about 17% (wet weight) while the final values ranging from 24.9% to 28.1%. At the end of the product maturation, the lipid percentage of samples inoculated with *L. sakei*/*S. xylosus* differed significantly (*p* < 0.05) in relation to the sausage diameter.

**Figure 4:**
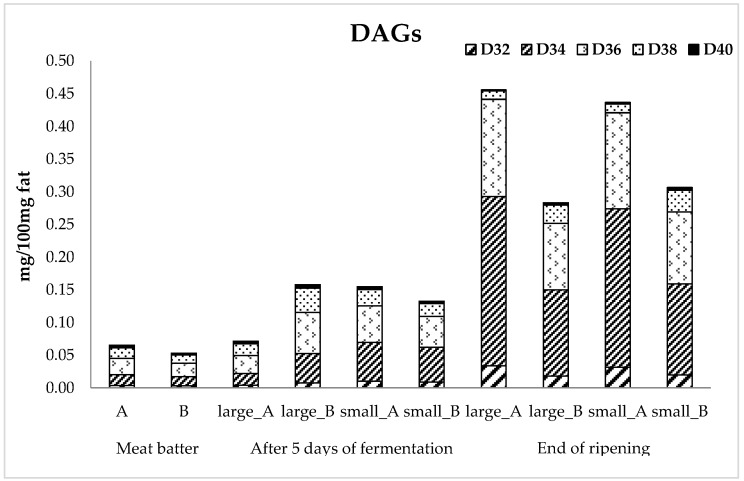
Principal DAG profile in the meat batter and in sausages inoculated with a different starter (*L. sakei*/*S. xylosus* and *P. pentosaceus*/*S. xylosus*) and with different diameters (large and small) during fermentation and ripening.

Lipid hydrolysis was evaluated by diacylglycerols (DAGs) analysis. At the end of ripening, all samples presented a significant (*p* < 0.05) higher DAG amount with respect to meat batter and a higher DAG accumulation was found in the sausages inoculated with *L. sakei*/*S. xylosus* independently from sausage size. As shown in Figure 4, in the meat batter, the D34 (C16–C18 diacylglycerols), D36 (C18–C18 diacylglycerols), and D38 (C18–C20 diacylglycerols) were the principal DAG series. An evolution of these compounds was observed during ripening with a significant (*p* < 0.05) increase in D32 (C16–C16 diacylglycerols), D34, and D36 series, which shows higher values in the sausages inoculated with *L. sakei*/*S. xylosus* compared with those in which *P. pentosaceus*/*S. xylosus* was added, which is similar to that observed for the total DAG content. Table 3 shows the content (mg FAs/100 g fat) in samples of the different MUFA, SFA, and PUFA classes during ripening. Sausages inoculated with *L. sakei*/*S. xylosus* showed a significant (*p* < 0.05) decrease of MUFA content both after 5 days of fermentation and at the 100% of ripening with final values five-fold less than sausages with *P. pentosaceus*/*S. xylosus*. No differences were found comparing salami of different diameters.

Peroxide index did not show significant differences among samples with values ranging between 11.6 and 16.5 meq of O_2_/kg of fat. Otherwise, TBARS values presented an upward trend after five days of fermentation, which was followed by a significant (*p* < 0.05) decrease at the end of maturation.

### 3.5. Three-way ANOVA and Principal Component Analysis (PCA)

A three-way ANOVA was performed to investigate the potential interaction between factors (sausage size, starter cultures, and process time) on biogenic amine content, proteolysis, and lipid fraction (Table 4). The three variables and their interactions significantly affected the accumulation of biogenic amines in the samples. On the contrary, proteolysis data were significantly influenced only by ripening time except for troponin C, which was significantly dependent on starters and ripening time. These latter factors and their interaction affected lipid content, MUFAs, and DAGs.

**Figure 5:**
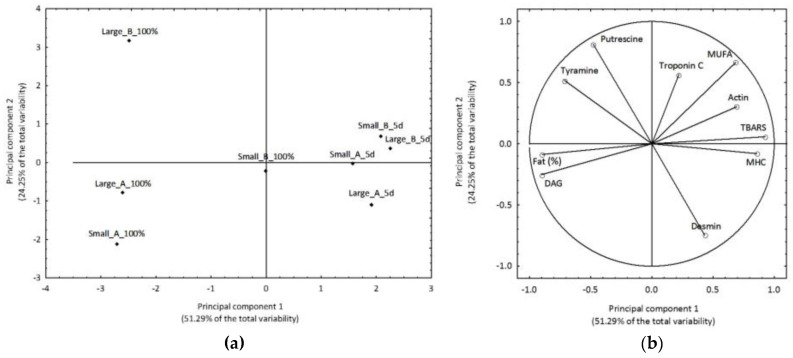
Case projection for the first two factors of large and small diameter sausages inoculated with starter cultures A (*Lactobacillus sakei*/*Staphylococcus xylosus*) or starter cultures B (*Pediococcus pentosaceus*/*Staphylococcus xylosus*) after fermentation and at the end of ripening (**a**) PCA loading plot of the variable selected on the first two factors obtained from PCA (**b**).

To further profile the samples and highlight the influence of diameter and starter on sausage characteristics during fermentation and at the end of ripening, a principal component analysis (PCA) was carried out by considering the results of biogenic amines, lipid fraction, and proteolysis. In particular, data showing significant differences (*p* < 0.05) were selected according to their discrimination power while variables that were redundant were not considered. The first two components explained more than 75% of the total variance, which included PC1 51.29% and PC2 24.25%. The resulting grouping of the sausage samples is shown in Figure 5a.

The samples collected after five days of fermentation are grouped on the right part of the graph and are slightly divided according to the starter on PC2. This was due to the weight of proteolysis variables known as TBARS and MUFAs (Figure 5b). As far as samples analyzed at the end of ripening, they are on the left side of the graph except for the small size sausage produced with *P. Pentosaceus*/*S. xylosus*, which is found on the origin of the axes. In this case, the effect of the different starter is well evidenced on PC2. In fact, sausages produced with *L. sakei*/*S. xylosus* are grouped in the third quadrant independently on the diameter. This separation was due mainly to lipid fraction and biogenic amine content (Figure 5b).

## 4. Discussion

Four batches of Milano-type dry fermented sausages were industrially produced with the aim to evaluate the effects of two different LAB starter cultures and diameter on BA content and sample ripening patterns during the production and at the end of maturation. Montanari et al. [25] evaluated the same samples as far as physico-chemical, microbiological, and aroma characteristics during processing and in the final products. It was found that results were influenced by the diameter in the staphylococcal population and the formation of volatile organic compounds. Nevertheless, the same authors reported that the choice of *L. sakei*/*S. xylosus* or *P. pentosaceus*/*S. xylosus* as starter cultures had a direct effect on the fermentation and the acidification rate. In fact, the sausages inoculated with *L. sakei*/*S. xylosus* showed a slower pH decrease during the fermentation and small sausages fermented by *L. sakei*/*S. xylosus* presented significant higher pH values (*p <* 0.05) with respect to those inoculated with *P. pentosaceus*/*S. xylosus* starter cultures at the end of ripening.

BA analysis revealed that tyramine and putrescine were the most important ones, as reported for these kinds of products [16,39]. The production of putrescine in food is often associated with the activity of Gram negative bacteria (pseudomonads and enterobacteria), but, in this case, the higher pseudomonads survival found in sausages inoculated with *P. pentosaceus*/*S. xylosus* [25] can only partially explain this diversity among samples. On the other hand, the production of putrescine by some Gram positive LAB has been demonstrated and these bacteria are considered as the main producers of BAs in fermented meat products [40,41]. Putrescine can directly derive from the decarboxylation of ornithine, which, in turn, is obtained by the metabolism of arginine (via the enzyme arginase). Alternatively, it can be obtained through the activity of a specific deiminase acting on agmatine (AgDI), which derives from arginine decarboxylation. This latter pathway has been demonstrated in several LAB including in *P. pentosaceus* and *L. sakei* [42,43]. Since starters are selected on the basis of their lack of aminobiogenic potential, the high putrescine content in a large sausage inoculated with *P. pentosaceus*/*S. xylosus* could be ascribed to the presence of some indigenous *L. sakei*, which has been previously highlighted by Montanari et al. [25]. These wild strains could produce putrescine from agmatine through the AgDI pathway, which produces ammonia toward acid stress due to the strong pediococci fermentation.

The tyramine presence in dry fermented sausages has been mostly related to the tyrosine decarboxylase activity of LAB [16]. Van Ba et al. [44] compared five different commercial starter cultures containing different LAB strains and their effects on the quality characteristics and BA contents in sausages. In agreement with our findings, these authors found that starter containing *P. pentosaceus*/*S. carnosus* led to a higher sausage tyramine content in comparison with *L. sakei*/*S. carnosus* in which the latter starter was more suitable for the production of high-quality products with lowered BA concentration.

BA content in samples differed in relation to the diameter as it has already been observed by several authors. Miguélez-Arrizado et al. [45] demonstrated that the tyramine accumulation in Spanish sausages was higher in salchichon (diameter 5–12 cm) rather than in the fuet (diameter < 3 cm) and tyramine was mainly accumulated in the central part of sausages. Similar results were reported by Komprda et al. [46] in typical Czech sausages and by Latorre-Moratalla [47] in fuet and llonganissa. In Italian salami, a similar trend was observed by Anastasio et al. [19] and, recently, Tabanelli et al. [21] found that BA amounts could be related to the size of sausages with the larger diameter including a higher concentration. The lower BA level in small size sausages can be attributed to the shorter ripening time and, consequently, to the time needed to reach a_w_ values able to inhibit the metabolism of decarboxylating microorganisms and/or the action of decarboxylases. Moreover, it has been demonstrated that the ability to produce BAs is negatively influenced by increasing concentration of NaCl [48,49,50]. In addition, the lower small size product acidity (which reduces the role of pH cell protective mechanism of decarboxylases) can limit BA accumulation. Some authors found higher tyramine production by *Lactococcus lactis* under anaerobic conditions [51]. This could be a supplementary reason for explaining the higher BA content in the sausages with a larger diameter, which caused a lower oxygen diffusion inside the product.

In the present study, the electrophoretic profiles of the myofibrillar proteins were investigated. On the other hand, being the main target for endogenous muscle peptidases [52], the sarcoplasmic protein fraction was not considered within this study.

Overall, the relative abundance of contractile (myosin and actin) and cytoskeletal proteins as well as of those polypeptide chains composing the tropomyosin complex profoundly changed during fermentation and ripening. In detail, the significant reduction at the end of ripening in the relative intensities of the bands ascribed to the main myofibrillar proteins (MHC, desmin, actin, tropomyosin, and troponin C) agreed with previous studies performed on dry sausages. A relevant decrease and even a complete degradation of MHC was observed during ripening [11,53]. In particular, since MHC had two regions target different proteases, the enzymatic hydrolysis of this protein led to several breakdown products with a wide range of molecular weight (50–150 kDa). In this context, MHC degradation was associated with a simultaneous appearance of a degradation product that has a molecular weight of 110 kDa. Similarly, the broad degradation of actin (42 kDa) agreed with previous investigations carried out to assess proteolysis during the processing of beef, pork, and horse-made fermented sausages [53,54,55]. Actin breakdown during ripening was widely demonstrated by electrophoresis [29] as well as more deeply investigated, resulting in the identification of its peptides by proteomic analysis through LC-MSE [7]. After ripening, the increased staining intensities of the electrophoretic bands ascribed to troponin T, troponin C, and tropomyosin might be the result of the co-migration of peptides derived from MHC and other high-molecular weight proteins [11,56].

Aside from the starter cultures, a diameter-effect on the amount of troponin C and proteolytic fragments was observed in salami inoculated with *L. sakei*/*S. xylosus*. This might be partially attributed to the different pH increase observed during ripening, which is mainly attributable to the faster respiration of lactic acid by molds. In fact, mold *hyphae* requires a higher time in large size sausages to penetrate inside the product due to the reduced oxygen availability within the mass [25]. The presence of two distinctive fragments (with molecular weight of 95.8 and 36.1 kDa) identified in salami inoculated with *L. sakei*/*S. xylosus* might be attributed to the exopeptidases activity and to the wide set of intracellular peptidases (such as endopeptidases, aminopeptidases, dipeptidases, and tripeptidases) found in *L. sakei.* These enzymes can be released as a consequence of cell lysis during fermentation, which leads to the formation of several polypeptides [52]. In the same time, the distinctive electrophoretic bands of 191, 174, and 150 kDa and the faster degradation of the polypeptide chains observed in salami inoculated with *P. pentosaceus*/*S. xylosus* likely resulted from hydrolysis of high-molecular weight proteins such as MHC, nebulin, vinculin, and titin. In addition, the accumulation of a 173 kDa degradation product was previously observed after nine days of processing in dry-fermented sausages [57].

Lipids are the major fraction of fermented sausages and they play a key role in their nutritional and sensory quality. Thus, the changes that occur to lipids due to lipolytic and oxidative phenomena during the ripening process were studied for these samples.

Diacylglycerols (DAGs) are a useful parameter to evaluate the extent of lipolysis and they are considered a more reliable parameter with respect to free fatty acids, which can easily react with other analytes of the system [58]. The increase in D32, D34, and D36 series during ripening in all samples could be due to the fact that pork fat is mainly constituted by fatty acids (FAs) with 16 and 18 carbon atoms [59] such as oleic (C18:1c9, ~50% of the total FAs), palmitic (C16:0, ~23%), stearic (C18:0, ~13%), and linoleic (C18:2, ~11%) acids (data not shown). The increase of the total DAG content and the decrease of MUFA class at the end of maturation in the sausages inoculated with *L. sakei*/*S. xylosus* could be related. Therefore, the starter culture with *L. sakei* seems to have a higher effect on lipolysis in fermented sausages than *P. pentosaceus* and these results reveal that the principal FAs losses for lipolysis were MUFA (oleic acid, as principal one), which was already evaluated in previous investigations [60].

Lipid oxidation was also analyzed in order to better describe the lipidic evolution in dry fermented sausages and evaluate their final quality. Peroxide index (PV) and TBARS values were respectively used as indices of primary and secondary products of oxidation and they were relatively high in all samples. Other studies already reported high values for oxidative parameters in fermented sausages [61,62], which might be because of the product manufacture: grinding and mixing of the meat increase the surface exposed to oxygen and oxidation catalysts [63]. However, these peroxide values were lower than 25 meq of O_2_/kg of fat, which is the limit of acceptability for fatty foods and did not show differences (*p <* 0.05) among the samples.

Moreover, this TBARS behavior was already observed in other research studies [61,62,64] because the peroxides, which are the primary oxidative products, degrade in malonaldehyde (MDA) and other reactive compounds. MDA reacts with amino acids, sugars, and nitrite during ripening and storage, which brings a decrease in TBARS.

## 5. Conclusions

The starter activity remarkably influenced an important safety aspect known as BA concentration. In particular, putrescine and tyramine were accumulated mostly in samples inoculated with *P. pentosaceus*/*S. xylosus*. This could be ascribed to the presence of some indigenous LAB and, consequently, to a non-optimal use of the starter culture. On the other hand, the adoption of the same environmental and processing conditions for sausages produced with different starters did not allow the strains to exert their best physiological aptitudes. For this reason, the choice of specific starter cultures and the optimization of their use and their performances, in relation to the product characteristics and process conditions, should be the key factor for improving safety and peculiarity of traditional fermented sausages.

## Figures and Tables

**Table 1:** Biogenic amine content (mg/kg) of the samples during fermentation and ripening.

Sampling Points ^1^	Starter Culture ^2^	Histamine	Tyramine	Putrescine	Cadaverine
Meat batter	A	- ^3^	- ^a^	- ^a^	- ^a^
	B	-	- ^a^	- ^a^	- ^a^
**Large size sausages**					
After 5 days of fermentation	A	-	- ^a^	1.74 ^a^	- ^a^
	B	-	0.71 ^a^	2.00 ^a^	0.58 ^a^
30% of ripening (38 days)	A	-	120.97 ^b^	2.87 ^a^	3.76 ^a^
	B	2.20	145.22 ^c^	40.14 ^b^	2.05 ^a^
60% of ripening (69 days)	A	-	142.63 ^c^	3.98 ^a^	9.02 ^b^
	B	-	155.67 ^d^	56.78 ^c^	7.26 ^b^
100% of ripening (105 days)	A	0.22	177.32 ^e^	8.68 ^a^	1.14 ^a^
	B	1.94	204.15 ^f^	128.17 ^d^	1.95 ^a^
**Small size sausages**					
After 5 days of fermentation	A	-	- ^a^	- ^a^	- ^a^
	B	-	- ^a^	- ^a^	- ^a^
30% of ripening (11 days)	A	-	1.67 ^a^	1.43 ^a^	1.15 ^a^
	B	-	11.73 ^g^	1.68 ^a^	0.89 ^a^
60% of ripening (17 days)	A	-	3.45 ^ag^	0.98 ^a^	1.59 ^a^
	B	-	9.89 ^g^	1.06 ^a^	0.83 ^a^
100% of ripening (28 days)	A	-	6.50 ^ag^	1.38 ^a^	2.48 ^a^
	B	1.75	9.09 ^g^	2.73 ^a^	0.95 ^a^

^1^ 30%, 60%, and 100% of ripening corresponded to 38, 69, and 105 days for large diameter sausages and 11, 17, and 28 days for small diameter sausages. ^2^ Starter cultures A (*L. sakei*/*S. xylosus*), starter cultures B (*P. pentosaceus*/*S. xylosus*). ^3^ Under detection limit (0.5 mg/kg). The values are the mean of three independent samples. Different superscript letters represent statistically significant differences (*p* < 0.05), according to Fisher’s LSD test of the ANOVA for each BA. When no pick has been detected, the value was set at zero. ”-“: not detected.

**Table 2:** Relative abundance (%) of the main myofibrillar proteins identified in the samples after fermentation and ripening. The values are the mean of three independent samples.

Sampling Points ^1^	Starter Culture ^2^	MHC (200 kDa)	Desmin (53 kDa)	Actin (42 kDa)	Troponin T (35.2 kDa)	Tropomyosin (34 kDa)	Troponin C (21.6 kDa)
Meat batter	A	7.5 ^a^	6.0 ^a^	8.8 ^a^	6.3 ^a^	5.6 ^a^	4.8 ^a^
	B	7.7 ^a^	5.7 ^a^	7.6 ^a^	5.9 ^a^	5.9 ^a^	5.2 ^a^
**Large size sausages**							
After 5 days of fermentation	A	7.6 ^a^	3.6 ^b^	4.5 ^b^	4.4 ^a^	- ^b^	- ^c^
	B	5.9 ^ab^	3.6 ^b^	5.8 ^b^	- ^b^	4.5 ^c^	4.1 ^ab^
100% of ripening (105 days)	A	3.7 ^b^	3.5 ^b^	3.6 ^b^	4.9 ^a^	5.0 ^ac^	3.1 ^b^
	B	3.6 ^b^	2.9 ^b^	4.2 ^b^	3.8 ^a^	3.2 ^c^	2.9 ^b^
**Small size sausages**							
After 5 days of fermentation	A	5.0 ^ab^	3.4 ^b^	3.9 ^b^	4.7 ^a^	4.7 ^c^	2.9 ^b^
	B	5.6 ^ab^	3.3 ^b^	5.5 ^b^	- ^b^	3.9 ^c^	3.4 ^b^
100% of ripening (28 days)	A	3.1 ^b^	3.4 ^b^	3.4 ^b^	4.2 ^a^	4.4 ^c^	- ^c^
	B	4.2 ^b^	3.6 ^b^	5.4 ^b^	4.4 ^a^	3.7 ^c^	3.5 ^b^

^1^ 100% of ripening corresponded to 105 days for large diameter sausages and 28 days for small diameter sausages.^2^ Starter cultures A (*L. sakei*/*S. xylosus*), starter cultures B (*P. pentosaceus/S. xylosus*), ”-“: not detected. The values are the mean of three independent samples. Different lower case letters represent statistically significant differences between samples for each myofibrillar protein (*p* < 0.05).

**Table 3:** Lipid (% *w*/*w*), fatty acids (mg/100 mg fat), diacylglycerols (g/100 mg fat), peroxide value (meq O_2_/kg fat), and TBARS (mg MDA/kg sample) of the samples during fermentation and ripening.

Sampling Points ^1^	Starter Culture ^2^	Lipid	MUFAs	PUFAs	SFAs	DAGs	PV	TBARS
Meat batter	A	16.95 ^a^	42.22 ^a^	10.83	32.60	0.06 ^a^	13.23	18.58 ^a^
	B	16.37 ^a^	35.49 ^ab^	10.74	26.83	0.05 ^a^	11.59	15.99 ^a^
**Large size sausages**								
After 5 days of fermentation	A	19.67 ^b^	29.42 ^b^	7.41	22.73	0.07 ^ab^	14.04	19.08 ^ab^
	B	15.36 ^a^	37.37 ^ab^	10.86	28.75	0.16 ^bc^	13.62	20.32 ^ab^
100% of ripening (105 days)	A	25.23 ^c^	6.63 ^c^	10.42	30.65	0.46 ^d^	13.56	12.52 ^c^
	B	26.14 ^cd^	35.31 ^ab^	10.35	27.87	0.28 ^e^	13.47	12.64 ^c^
**Small size sausages**								
After 5 days of fermentation	A	19.20 ^b^	31.13 ^b^	8.08	24.06	0.15 ^bc^	16.48	22.35 ^ab^
	B	17.48 ^ab^	33.21 ^ab^	9.12	25.76	0.13 ^bc^	13.84	21.81 ^ab^
100% of ripening (28 days)	A	28.12 ^d^	5.24 ^c^	8.81	26.12	0.44 ^d^	14.45	12.14 ^c^
	B	24.86 ^cd^	31.56 ^b^	9.04	24.51	0.31 ^e^	13.72	13.76 ^c^

^1^ 100% of ripening corresponded to 105 days for large diameter sausages and 28 days for small diameter sausages. ^2^ Starter cultures A (*L. sakei*/*S. xylosus*), starter cultures B (*P. pentosaceus*/*S. xylosus*), MUFAs, monounsaturated fatty acids, PUFAs, polyunsaturated fatty acids, SFAs, saturated fatty acids, DAGs, diacylglycerols, PV, peroxide value, TBARS, Thiobarbituric Acid-Reactive Substances. The values are the mean of three independent samples. Different superscript letters represent statistically significant differences (*p* < 0.05), according to Fisher’s LSD test of the ANOVA for each lipid fraction.

**Table 4:** Influence of factors on biogenic amines, proteolysis, and lipid fraction using three-way ANOVA. A: Sausage size. B: Starter cultures. C: Ripening time (sampling point).

Variables	A	B	C	A × B	A × C	B × C	A × B × C
Tyramine	**	**	**	**	**	**	-
Cadaverine	**	**	**	-	**	*	**
Putrescine	**	**	**	**	**	**	**
MHC	-	-	*	-	-	-	-
Desmin	-	-	-	-	-	-	-
Actin	-	-	*	-	-	-	-
Troponin T	-	-	*	-	-	-	-
Tropomyosin	-	-	**	-	-	*	*
Troponin C	-	**	**	-	-	-	**
Lipid	-	**	**	-	-	-	*
MUFAs	-	**	**	-	-	**	-
PUFAs	-	-	-	-	-	-	-
SFAs	-	-	-	-	-	-	-
DAGs	-	*	**	-	-	**	-
PV	-	-	-	-	-	-	-
TBARS	-	-	*	-	-	-	-

* and ** represent that the factors or their interactions significantly affect the results at *p* < 0.05 and *p* < 0.01, respectively, while “-” means that the factors or their interaction have no significant effect (*p* > 0.05).
